# Alcohol use during COVID-19 pandemic on the long run: findings from a longitudinal study in Germany

**DOI:** 10.1186/s40359-022-00965-8

**Published:** 2022-11-14

**Authors:** Henrike Schecke, Annette Bohn, Norbert Scherbaum, Christian Mette

**Affiliations:** 1 Department of Psychiatry and Psychotherapy, Medical Faculty, LVR-Hospital Essen, University of Duisburg-Essen, Essen, Germany; 2 Department of Social Work and Education, Department of Psychology, Protestant University of Applied Sciences Bochum, Bochum, Germany

**Keywords:** COVID-19, Longitudinal design, Alcohol use, Substance use

## Abstract

**Background:**

The impact of COVID-induced stress on mental health and alcohol use has been demonstrated in recent research. However, there is a lack of longitudinal data since most studies reported on cross-sectional data. It remains unclear how alcohol use develops under the dynamic changes of the pandemic. Therefore, the present study aims to investigate the general development of alcohol use and the impact of COVID related stress on drinking behavior in a German population-based sample during the pandemic in 2020.

**Methods:**

In the longitudinal design with three measurements (baseline [T1] and two follow-ups [T2, T3]) an online survey was administered. The survey included the Alcohol Use Disorder Identification Test (AUDIT) as well as the assessment of the drinking days in the last 30 days, the number of alcoholic beverages on each occasion and changes in alcohol use in the previous fourteen days. Further, COVID-19 related concerns, perceived stress, worries about friends and family and worries about the financial situation were also assessed and multiple linear regressions and confidence intervals (CI) were calculated.

**Results:**

1050 participants started the survey, 756 participants (71.4%), 317 (52.7%) participants completed the survey at all three measurements. Seventy six percent (n = 241) of the sample were female. An increase in alcohol use in the previous 14 days was reported by 10.9% at T1, 3.9% at T2 and 3.6% at T3. Moreover, a decrease in alcohol use in the previous 14 days was reported by 8.7% at T1, 6.5% at T2 and 4.1% at T3. The number of drinking days was significantly higher at T2 than at baseline (*p* < .001; F = − 2.06, [CI − 3.10, − 1.02]). COVID-19 related concerns and stress were associated with a higher number of drinking days and average number of drinks at a typical occasion. AUDIT score at baseline positively predicted number of drinking days as well as average number of drinks.

**Conclusions:**

The significant increases in alcohol use is a public health issue during COVID-19 pandemic. The findings show that especially people who drink more hazardously previously tend to drink more under pandemic conditions. Those individuals are particularly at risk for developing substance-related problems.

## Introduction

The outbreak of the COVID virus poses new challenges for societies worldwide [1]. Governments in various countries around the world responded to the pandemic outbreak and its rising incidence with social distancing policies and lockdowns. However, these restrictions led to an increase in stress, which had a negative impact on health and psychological conditions [2]. Other stressors to be mentioned are the fear of infection and its severe course, the fear of close relatives suffering from COVID-19 and also the economic consequences, such as short-time work or job loss [3–5].

Recent research showed that the COVID-19 situation negatively affects mental health. Depressive symptoms, anxiety or sleep disturbances increased [1–6]. Distress, anxiety and depressive symptoms are well described risk factors for increases in substance use. Alcohol use as a coping mechanism for COVID-19-related stressors has been the focus of recent research activities on psychosocial burdens of COVID-19 [7–9]. A steadily increasing number of publications document changes in alcohol consumption under COVID-19 pandemic conditions for different countries, e.g. for the United States [7], the United Kingdom [10], Poland [11], Australia [12], Germany [13] or France [14]. These studies have shown that between one fifth and one quarter of adults increased alcohol use after the advent of the COVID-19 crisis. However, in some studies, a comparable proportion also reported a reduction in alcohol consumption. This may be a consequence of the social distancing policy and lockdown measures, since the opportunity to drink in larger groups, e.g., in bars, restaurants or at parties, was eliminated. Contrasting previous findings, a recent study in 21 European countries showed that in most countries, except for Ireland and the United Kingdom, there was a decrease in alcohol consumption. However, a reduction in alcohol consumption was less common among people who were particularly stressed by the pandemic [15].

Most studies in the field of COVID-19 and substance use focus on non-clinical populations, but COVID-19 related stressors could also have a particularly negative impact on individuals living with alcohol use disorders (AUD). In a retrospective clinical study, increases in relapse rates among adults living with AUD were observed during the pandemic. Depression, anxiety and living alone during COVID-19 were major risk factors for relapses among individuals with AUD [16].

The impact of COVID-induced stress on mental health and alcohol use has been demonstrated in recent research. However, there is a lack of longitudinal data since most studies reported on cross-sectional data. It remains unclear how alcohol use develops under the dynamic changes of the pandemic. Therefore, the present study aimed to investigate how alcohol use in a German population-based sample developed over time in the course of the pandemic in the year 2020. In addition, the extent to which COVID-19-related stressors influenced alcohol use was assessed.

We therefore hypothesized that there was an increase over time in the use of alcohol, operationalized by the number of drinking days and the number of drinks.

Furthermore, we hypothesized that there was a positive correlation between COVID-19 related stress and COVID-19 related concerns and the number of drinking days and the number of drinks. In addition, the AUDIT score at T1 is a positive predictor of the number of drinking days and the number of drinks.

## Material and methods

The study design was a longitudinal, with a baseline measurement (T1) and two follow-up measures (T2, T3), using a self-completed online survey with a self-selected convenience sample from the German general population. All participants had the opportunity to answer anonymously but were asked to submit an e-mail address to be contacted for two follow-up online surveys. Participants received a personal invitation to participate at the follow-up surveys via e-mail, which included a personalized link. The personal e-mail address was deleted immediately from the dataset after the last follow-up. Participants were able to withdraw from the study at any time. The initial baseline (T1) survey started in March 2020, including the very early stage of the pandemic and the first period of COVID-19 related contact restrictions in Germany. The follow-up surveys were administered in June 2020 and November 2020. At the first follow-up (T2), the COVID-19 incidence in Germany was quite low and contact restrictions were less strict than during baseline period and the second follow-up (T3), which comprises a period of high incidence rates and with re-establishment of contact restrictions before the Christmas holiday’s season.

Inclusion criteria were at least 18 years of age and German language capabilities, since the survey was only available in German. Participants were recruited via social media, institutional newsletters, and online press releases. Commercial advertisements or paid social media postings were not used. There was no financial compensation or other incentives for participation.The open source *lime survey tool* was used for the online data collection.

The study was conducted in accordance with the Declaration of Helsinki, and the Ethics Committee of the University Hospitals Essen has approved the study (Protocol code: 21-10325-BO). All participants signed an informed consent prior to study inclusion.

The socio-demographic characteristics were assessed once at the initial survey.

At all measures alcohol use in the previous 30 days and changes in alcohol and/or cigarette use in the previous 14 days were assessed. To screen for alcohol related disorders the Alcohol Use Disorder Identification Test (AUDIT) [17, 18] was administered once. The AUDIT is a standard screening instrument for assessing AUD. It is a valid and reliable instrument and has sufficient psychometric properties (intra-class-coefficient total score = .95; retest reliability kappa = .81–.86, sensitivity and specificity AUD .97 and .92). No additional psychometric parameters were calculated for the present sample.

Further, COVID-19 related items were assessed in the survey. Because the survey was designed and conducted within the first few weeks of the COVID-19 pandemic, validated instruments were not available at this early stage. For this reason, the author group developed an ad hoc questionnaire with COVID-19 relevant items. These items included questions about COVID-19 related aspects regarding the occupational situation (e.g. job loss, working from home), increased care activities for children or older people and changes in social and recreational activities. In addition, COVID-19 related concerns, perceived stress, worries about friends and family and worries about the financial situation were assessed. Thoseitems used an eleven-point Likert-Scales (0 = not at all until 10 = very strong).

Data analysis was conducted using IBM SPSS Statistics 27 software. The preliminary assumptions were checked and met for parametric analyses (e.g. normality, homogeneity of variances) as well as for multiple regression analysis (e.g. independent residuals, homoscedasticity, no multicollinearity). Multiple linear regressions were carried out with the number of days with alcohol consumption in the previous 30 days on each measurement and the number of glasses on each drinking occasion on each measurement as the outcome variables. As predictors, participants’ COVID-19 related stress and concerns on each respective measurement as well as the AUDIT score were included in the analyses. Further, confidence intervals (CI) were calculated. Table 4 shows all regression models with their significant predictors. To compare the number of days with alcohol consumption in the previous 30 days on each measurement and the number of glasses of alcohol that were consumed on each drinking occasion a repeated measure analysis of variance was used (RMANOVA). All RMANOVAS were corrected by using post hoc overall Bonferroni correction and, if necessary, Greenhouse Geiser adjustment. Effect sizes are stated by Cohen’s f and were calculated with partial η2, which estimates the proportion of variance in the dependent variable that is attributable to the group effect. We interpreted the effect sizes according to Cohen (η2 = .01 [small effect], η2 = .06 [medium effect] and η2 = .14 [large effect]) [19]. The significance level was set at *p* < 0.05.

## Results

### Sample

At baseline (T1), 1050 participants started the survey, 756 participants (71.4%) completed all questions and of those 610 participants (80.7%) submitted an e-mail address for the follow-ups. 317 (52.7%) participants completed the survey at all three measurements. A drop out analysis revealed that non-completers were significantly more often between 25 and 54 years of age (*p* = .047), were less often students or retired persons (*p* = .024) and reported a lower number of drinking days in the last 30 days (6.9 vs. 8.6, *p* = .044) at baseline measurement. All further statistical analyses refer to the sample of those 317 completers. Seventy six percent (n = 241) of the sample were female and highly educated. Over 80% had the highest school diploma or a university degree, 84% were employees or students. About one quarter were single and/or lived alone. Two third of the sample lived in a flat or house which had 70 to more than 100 square meters (Table 1).

**Table 1:** Sociodemographic characteristics of sample that completed all measurements (N = 317)

Variable	N	%
Gender		
Female	241	76
Male	76	24
Age		
18–24	37	11.7
25–39	126	39.7
40–54	89	28.1
55–65	47	14.8
65 and older	18	5.7
Education		
Certificate of secondary education	3	.9
General certificate of secondary education	31	9.8
University entrance diploma	100	31.5
University degree	183	57.7
Occupation		
Employed	203	64
Student	65	20.5
Apprentice	3	.9
Retired	20	6.3
Other	15	4.7
Selfemployed (exclusivley)	11	3.5
Relationship status		
Single	81	25.6
In a relationship, living apart	40	12.6
In a relationship, living together	196	61.8
Size of flat/house		
< 30 m^2^	3	.9
30–50 m^2^	32	10.1
50–70 m^2^	56	17.7
70–100 m^2^	99	31.2
> 100 m^2^	127	40.1
Living situation		
Living alone	78	24.6
Living with partner	112	35.3
Ling with partner and children	71	22.4
Living with other people	56	17.7

### COVID-19 related items

On average, COVID-19 related stress and concerns ranged around five on the eleven-point Likert-scale at all measures. Worries about own health increased over time, from around 3 to 4 on average, but were consistently lower than worries about friends and family which ranged around 6 points on the Likert-scale. Financial concerns were low on average, ranging around 2 at all measures. Details regarding COVID-19 related items are presented in Table 2.

**Table 2:** COVID-19 related variables on all three measurements (N = 317)

Variable	T1						T2						T3					
	All		♂		♀		All		♂		♀		All		♂		♀	
	N	%	N	%	N	%	N	%	N	%	N	%	N	%	N	%	N	%
COVID related changes occupation																		
Working from home	143	45.1	31	40.8	112	46.5	137	43.2	31	40.8	106	44	117	36.9	28	36.8	89	36.9
Short-time work	15	4.7	3	3.9	12	5.0	18	5.7	4	5.3	14	5.8	7	2.2	2	2.6	5	2.1
Job loss	4	1.3	1	1.3	3	1.2	5	1.6	1	1.3	4	1.7	9	2.8	4	5.3	5	2.1
Loss of freelance work	45	14.2	14	12.9	31	12.9	45	14.2	17*	22.4*	28*	11.6*	28	8.8	9	11.8	19	7.9
COVID related changes care activities																		
Children < 12	49	15.5	9	11.8	40	16.8	45	15.5	10	13.2	39	16.2	14	4.4	2	2.6	12	5.0
Elderly persons	73	23	18	23.7	55	22.8	62	19.6	13	17.1	49	20.3	56	17.7	13	17.1	43	17.8

	M	SD	M	SD	M	SD	M	SD	M	SD	M	SD	M	SD	M	SD	M	SD
COVID concerns	5.49	2.44	4.87	2.41	5.69	2.43	4.61	2.35	4.45	2.46	4.66	2.33	5.63	2.22	5.30	2.26	5.73	2.20
COVID stress	5.28	2.75	4.55	2.81	5.51	2.70	5.16	2.57	4.71	2.60	5.31	2.55	5.79	2.46	5.16	2.46	6.00	2.43
Worries own health	3.56	2.6	3.28	2.34	3.65	2.67	3.15	2.40	2.89	2.32	3.23	2.42	4.48	2.55	4.09	2.44	4.61	2.58
Worries health others	6.9	2.32	6.29	2.47	7.10	2.24	6.14	2.47	5.54	2.53	6.33	2.42	6.87	2.28	6.30	2.36	7.05	2.23
Worries finances	2.75	2.85	2.21	2.47	2.91	2.94	2.3	2.56	2.08	2.51	2.37	2.58	2.28	2.63	2.01	2.59	2.37	2.65

*Chi^2^-Test showed significant differences (*Χ*^2^ = 5.482, *p* = .019, df 1). All other measures did not differ significantly between men and women

For all five items, RM ANOVAS were calculated to compare the mean scores between T1, T2 and T3 each. In every model gender was included as a between-subject factor. The main effect for all models showed to be significant, while the between-subject-factor did not show to have a significant effect in any model. All model’s results for the main effects, their effect sizes, as well as results from their post-hoc-tests can be found in Table 3.

**Table 3:** Results for repeated measure ANOVAs comparing T1, T2 and T3 for Covid-19 related variables each

Model’s main effect	df	df E	F	*p*	η_p_^2^	f	M difference T1–T2	M difference T2–T3	M difference T1–T3
Covid concerns	2	630	24.025	< .001*	.071	.28	.727*	− .967*	− .240
Covid stress	2	630	7.663	.001*	.024	.16	.023	− .568*	− .545*
Worries own health	1.86	590.74	51.48	< .001*	.140	.40	.398*	− 1.285*	− .887*
Worries health others	2	630	22.324	< .001*	.066	.27	.759*	− .743*	.016
Worries finances	1.78	560.1	4.42	.012	.014	.11	.335*	.037	.373

*p < .05

### Substance use

Over all measures, current tobacco use was reported by 10.7–11.7% of the sample. Alcohol use was reported by 58–63.4%. Fifteen percent reached an AUDIT score about the cut-off for hazardous drinking. An increase in alcohol use in the previous 14 days was reported by 10.9% at T1, 3.9% at T2 and 3.6% at T3 (Table 3). A decrease in alcohol use in the previous 14 days was reported by 8.7% at T1, 6.5%, and 4.1% at T2 and T3 respectively. All descriptive data on substance use are displayed in Table 4.

**Table 4:** Substance related variables on all three measurements

Variable	T1						T2						T3					
	All		♂		♀		All		♂		♀		All		♂		♀	
	N	%	N	%	N	%	N	%	N	%	N	%	N	%	N	%	N	%
Current nicotine use	34	10.7	8	10.5	26	10.7	34	10.7	9	11.8	25	10.3	37	11.7	10	13.2	27	11.2
Current alcohol use	184	58	49	64.5	135	56	201	63.4	52	68.4	149	61.8	193	60.9	52	68.4	141	58.5
Changes alcohol last 14 days																		
Increase	20	10.9	5	10.2	15	11.1	8	3.9	3	5.7	5	3.4	7	3.6	2	3.8	5	3.5
Decrease	16	8.7	4	8.1	12	8.9	13	6.5	2	3.8	11	7.4	8	4.1	2	3.8	6	4.3
Main setting alcohol use																		
Friends	102	55.4	23	46.9	79	58.5	112	55.7	20	38.5	92	61.7	88	45.6	16	30.7	72	51
Family	68	37	20	40.8	48	35.6	71	35.3	22	42.3	49	32.9	88	45.6	27	51.9	61	43.3
Alone	14	7.6	6	12.2	8	5.9	18	9	10	19.2	8	5.4	17	8.8	9	17.3	8	5.7
AUDIT Score																		
< 8	156	84.8	37	75.5	119	88.1												
> = 8	28	15.2	12	24.5	16	11.9												

	M	SD	M	SD	M	SD	M	SD	M	SD	M	SD	M	SD	M	SD	M	SD
Days alcohol use last 30 days	8.17	7.47	10.1	8.67	7.47	6.88	9.37	8.18	11.6	9.46	8.7	7.56	8.39	7.68	10.5	9.03	7.64	7.05
Average number glasses alcohol per occasion	2.15	2.15	2.74	3.19	1.95	1.64	1.86	1.29	2.25	1.59	1.74	1.14	2.28	2.95	2.67	3.13	2.14	2.89
AUDIT Score	4.9	4.5	6.02	4.39	4.5	2.97												

RMANOVA showed that the number of days with alcohol use in the previous 30 days differed significantly between measurements (F = (1.92, 306.00) = 9.69, *p* < .001, partial η^2^ = .057). The Bonferroni-adjusted post-hoc analysis demonstrated a significant difference (*p* < .001) between the number of days on T1 and T2 (− 2.06, 95%-CI [− 3.10, − 1.02]), indicating that the number of days with alcohol consumption on T2 had been higher than on T1. All other comparisons did not show a significant difference.

RMANOVA revealed that the average number of glasses of alcohol that was consumed on each drinking occasion did not differ significantly between measurements (F = (1.61, 243.55) = 1.27, *p* = .278).

For the number of days with alcohol consumption, the models’ adjusted R^2^ ranged between .042 and .116, showing a low to moderate goodness of fit. The models’ goodness of fit decreased from T1 to T3. Worries concerning the health of friends and family showed to be a negative predictor on all three measurements. COVID-19 related concerns were a positive predictor on T1 and T2, while the AUDIT score positively predicted the number of drinking days on T1 and T2.

The models’ adjusted R^2^ for the glasses of alcohol consumed on each drinking occasion showed a moderate goodness of fit for T1 and T2 with .196 and .197 respectively, and a low goodness-of-fit for the third model with R^2^ = .061. While the AUDIT Score was a positive predictor on all three measurements, only on T2 COVID-19 related stress showed to be a positive predictor and COVID-19 related concerns a negative predictor for the average number of glasses (Table 5).

**Table 5:** Overview of all multiple linear regression models

Model and predictors	R^2^	B	SE B	Beta	*p*
Number of days with alcohol use previous 30 days					
T1	.116				
Concerns Covid T1		.649	.304	.209	.034
Worries health of friends and family T1		− .765	.254	− .224	.003
AUDIT Score		.645	.152	.299	< .000
T2	.062				
Worries about health of friends and family T2		− .670	.279	− .206	.017
AUDIT Score		.436	.174	.182	.013
T3	.042				
Concerns Covid T3		1.113	.398	.306	.006
Worries health of friends and family T3		− .659	.303	− .206	.031
Average number of alcoholic beverages on each occasion					
T1	.196				
AUDIT Score		.280	.042	.459	< .000
T2	.197				
Concerns Covid T2		− .148	.056	− .264	.009
Stress Covid T2		.111	.039	.219	.007
AUDIT Score		.150	.025	.396	< .000
T3	.061				
AUDIT Score		.294	.080	.297	< .000

## Discussion

The study investigates the effects of COVID-19 on alcohol use with a longitudinal design in a German sample over a nine-month period of the pandemic in 2020. In the early stage of the pandemic in spring 2020, one in ten reported to drink more alcohol in the previous 14 days. This rate was lower at the second and third measure. Here, around 4% reported to drink more alcohol in the last 14 days. However, the number of drinking days was significantly higher at the second measure in summer 2020 than at baseline. The quantity of alcohol used on a typical occasion did not differ significantly between all measures. COVID 19 related concerns regarding friends’ and families’ health were associated with less alcohol use. COVID-19 related concerns and stress were associated with a higher number of drinking days in the last month and average number of drinks at a typical occasion. The AUDIT score at baseline positively predicts number of drinking days as well as average number of drinks.

The proportion of those who increased their alcohol consumption in the course of the COVID-19 pandemic in this study was considerably lower than that from previous cross-sectional studies, which consistently showed an increase of about one-quarter during the first wave of COVID-19 [7, 10–12]. Data analysis from the “Global Drug Survey” during COVID-19 even revealed an increase in alcohol use of more than thirty percent [20]. Despite a very large sample (N > 55,000), those data are not representative, since participants are predominately young and tend to be more affine and experienced with various substance use than other individuals in the general population. A recent study in different EU-countries found an overall reduction in alcohol use in most EU-countries [15]. Recent research indicates that some subpopulations increase their substance use more than others. During the COVID-19 lockdowns, women, parents of young children, middle-age people, people with higher socioeconomic status and individuals with depressive and anxiety symptoms reported the highest increase in alcohol consumption, for example in Australia, Belgium, France, the United Kingdom and the United States [21].

In the context of these results, it seems unexpected that no greater increase in alcohol consumption was observed in the study population of the present study. The sample consisted of more than two third of women, in the age group of young and middle-aged adults, one fifth lived with children and educational level and occupational situation indicate a higher socioeconomic status. Furthermore, in Germany among women, alcohol consumption increases with higher socioeconomic status [22]. Women with a higher socioeconomic status are twice as likely to drink hazardously than women with a middle or low socioeconomic status [22]. Against this background, a more significant increase in alcohol consumption would not have been unexpected. Financial and existential concerns could form a moderating effect. Kilian et al. (2021) showed that in the group of persons with high socioeconomic status, drinking was only increased when stress and financial worries were present [15]. In the present sample, the vast majority was not affected by job-loss or short time work due to COVID-19, so existential concerns were not likely. Other sample characteristics may also help to explain why the increase in alcohol consumption appeared less pronounced. In our study sample, a higher proportion were alcohol abstainers than in representative samples in the German general population (36.3–42% versus 28.4%) [23]. It can be assumed that individuals who never drink alcohol will also not start to use alcohol as a coping strategy for stress or reduced well-being during the pandemic.

Previous studies [24] argued that consequences of social distancing policies promote increases in alcohol use. Social distancing and self-isolation came along with disruption of daily routines, boredom, loss of daily structure and lack of social contacts which were identified as motives for a rise in alcohol consumption during the pandemic [24]. An US-study [25] also found that the longer people spent time at home alone, the higher the risk of binge-drinking at home [25].

Most participants in the present study reported drinking alcohol primarily in social situations. Against this background, the most significant increase in alcohol consumption at T1, during the first "lockdown," is remarkable, since the opportunities for gatherings in larger groups were clearly limited here. Coherent with the aspect of social drinking was that the number of drinking days and quantity were highest at T2. In summer 2020 contact restrictions were lowest, which opened up more opportunities to experience social encounters with friends or family and to drink alcohol in a social setting. In addition, the vast majority of study participants did not live alone, so social isolation and loneliness may have been less of a concern.

A further motivator to drink more alcohol might be decrease of negative emotions and stress. Recent studies found out that the COVID-19 pandemic negatively affects mental health [1–3, 26]. Studies demonstrated that depressive symptoms, anxiety, and psychological distress increased as a response to COVID-19 in Germany [4, 5]. Furthermore, repeated cross-sectional studies have shown that the implementation of contact restrictions was associated with increased levels of depression and anxiety. This level of depression and anxiety appears to persist even when these restrictions are removed [27]. However, psychological stress and mental health problems are well-characterized risk factors for the increase in the use of alcohol and other substances. For COVID-19-specific fears, the study evidence is unclear. The reason for this might be that specific scales were rare in the early phase of the pandemic. The present survey differentiated between COVID-19-related stress, COVID-19 related concerns, worries about friends’ and family’s health, worries about own health and worries about the own financial situation. With respect to drinking frequency, the higher worries about family/friends were, the less often alcohol was drunk. General COVID-19 related concerns and stress were positive predictors of drinking days at the first two measures. The latter findings underline the hypothesis that coping with stress and worries might be a relevant motivation to increase alcohol use, which was shown in previous cross-sectional studies [7, 8, 28].

For consumed amount of alcohol on each occasion, the findings were indifferent at the different points in time. At the second measurement, COVID-19 related stress positively predicted the number of glasses of alcoholic beverages, but COVID-19 related concerns negatively predicted quantity measures. These findings contrast with a study among US-women. Here, psychological distress related to COVID-19 was significantly associated with quantity measures of alcohol use, like the number of drinks at the last heaviest drinking event and the number of drinks on a typical occasion [7]. In contrast, perceived threat of the virus itself was not associated with more alcohol use [7]. Whereas among Russian, Belarusian and Israeli students COVID-19 related fear were significantly associated with increases in substance during the first wave of COVID-19 [29, 30].

Besides potential negative effects for individuals, significant increases in alcohol use are a public health issue. Hazardous alcohol use is associated with high burden of disease for individuals and societies [31]. In the present longitudinal study, the increase in alcohol consumption over the course of the pandemic was not as pronounced as at the beginning, but it was ascending slightly at all measurement time points. Especially for individuals, who are near the threshold for hazardous alcohol use, a constant rise in alcohol use in the course of one year might be of concern. The finding that the initial AUDIT score predicted drinking days and quantities underpins that especially people who drink more hazardously previously tend to drink more under pandemic conditions. Those individuals are particularly at risk for developing substance-related problems. In this vulnerable group, there is a possibility that an increase in psychological stress and coping attempts with alcohol may favor the development of an alcohol-related disorder.

Moreover, it may be discussed that pandemic control measures have led to a reduced availability of alcohol. However, this seems unlikely for Germany. Although bars, restaurants and clubs were closed, alcoholic beverages can be purchased in Germany at any grocery store, gas stations and kiosks that were also open during the "lockdowns".

It remains to be seen whether future epidemiologic studies of substance use in the general population will show a sustained increase in alcohol consumption or whether the increase under COVID-19 conditions merely deflect a short-term response to an exceptional situation. A sustained increase in alcohol consumption would be critical for a country like Germany, which has a high per capita alcohol consumption [32].

### Limitations

This study presents data from a population-based longitudinal survey covering different phases of the COVID-19 pandemic in Germany in 2020. This fills a gap in the state of research, as most studies to date have used a cross-sectional design. Nevertheless, some limitations need to be kept in mind. An online survey was used to collect data, thus, the possibility of selection bias and lack of representativeness needs to be considered. In addition, results might be biased in terms of socioeconomic status since high socioeconomic status was overrepresented. Furthermore, gender proportions were not balanced, and women were overrepresented. Regarding the measures used on COVID-19 specific fears, stress, and concerns, it is limiting to say that these included only one item each and were created ad hoc by the study group. This can be attributed to the fact that at the beginning of the study, in the early stage of the pandemic, COVID-19 specific scales were not yet available.

## Conclusions

In the future, pandemic response efforts should also consider mental health aspects and prevention of side effects such as an increase in substance use. It may be useful to focus on secondary prevention of alcohol use in vulnerable groups at an early stage. One approach may be e-mental health services and digital prevention campaigns for at-risk groups of problematic substance use. Policy makers should put prevention and counseling on risky alcohol use in the pandemic as a public health issue on their agenda. In addition, adequate financial resources must be made available if the need for treatment increases in the future.

## Data Availability

Due to ethical restrictions related to participant consent imposed by the Ethics Committee of the University of Duisburg Essen and the North-Rhine Westphalia Data Protection Act, all relevant data are available upon request to Dr. Christian Mette.
